# The Combination of Jiedu Xiaoluo Decoction with Autologous Peripheral Blood Stem Cell Transplantation (APBSCT) Accelerates Disease Remission of Non-Hodgkin Lymphoma

**DOI:** 10.1155/2021/2745705

**Published:** 2021-01-08

**Authors:** Yu Zhang, Jingjing Xiang, Ni Zhu, Hangping Ge, Xianfu Sheng, Shu Deng, Junfa Chen, Lihong Yu, Yan Zhou, Jianping Shen

**Affiliations:** ^1^Department of Hematology, First Affiliated Hospital of Zhejiang Chinese Medical University, Hangzhou 310000, China; ^2^First Medical College of Zhejiang Chinese Medical University, Hangzhou 310000, China

## Abstract

**Objective:**

This study aimed to explore the therapeutic effects of autologous peripheral blood stem cell transplantation (APBSCT) with Jiedu Xiaoluo decoction (JDX) on non-Hodgkin lymphoma (NHL).

**Method:**

B lymphoma cells A20 were used to establish nude mice-transplanted tumor model. The peripheral blood of mice was analyzed by automatic blood cell counter. Inflammatory cytokines in tumor tissues were measured by ELISA, real-time qRT-PCR, and western blotting assays. Immunohistochemical staining was employed to evaluate tumor cell growth and apoptosis. CCK8 and Transwell assays were used to detect cell viability, migration, and invasion. Cell apoptosis in vitro was evaluated with flow cytometry.

**Result:**

In the in vitro co-culture system of A20 cells and hemopoietic stem cells (HSC), JDX notably inhibited the proliferation, migration, and invasion and promoted apoptosis of A20 cells compared to HSC treatment alone. In animal tumor xenografts of NHL, the combination of APBSCT with JDX significantly promoted hematopoietic reconstitution, inhibited tumorigenesis of A20 cell, promoted the inflammatory microenvironment remission, inhibited cell proliferation, and promoted apoptosis compared to APBSCT alone.

**Conclusion:**

The combination of APBSCT with JDX might be an effective strategy to treat NHL through inhibiting tumorigenesis and reconstructing hematopoietic and immune microenvironment. Our finding provided a novel insight into the clinical application of Traditional Chinese Medicine (TCM) against NHL.

## 1. Introduction

Non-Hodgkin lymphoma (NHL) is a lymphoid malignancy with diverse biological and clinical behavior, including persistent painless lymphadenopathy or constitutional symptoms of other organs besides the lymphoid and hematopoietic system [1]. NHL is the third most common malignant tumor, accounting for ∼10% of all types of cancers [2]. NHL patients commonly receive chemoimmunotherapy as initial treatment such as high-dose chemotherapy combined with autologous peripheral blood stem cell transplantation (APBSCT) [3]. However, the controls of complications and long-term recurrence are still difficult due to the delayed hematopoietic recovery [4]. Hematopoietic stem cell (HSC) mobilization via regulating tumor microenvironment (TME) is considered as an important approach to control NHL, but mobilization agents of HSC are rare and often have strong side effects [5]. Currently, because the effects of traditional western medicine treatment are far from satisfactory to limit the process of hematological malignancies, more attention is being paid to potential roles for Traditional Chinese Medicine (TCM) in cancer treatment [6]. Xihuang pill (XH), as a common TCM, has strong clinical effects against NHL through regulating TME [7]. Dihydrocelastrol (DHCE), a dihydro-analog of celastrol isolated from *Tripterygium wilfordii* (the traditional Chinese medicinal plant), exerts potent anti-tumor activity in B-cell NHL through inhibiting mammalian target of rapamycin complex mTORC1 and mTORC2 and the downstream immune signaling transduction [8]. Chinese herb decoctions can modify the metastatic and inflammatory microenvironment of cancers including hematological neoplasms, resulting in tumor suppression [9]. These data indicate that TCM possibly acts as a immunologic adjuvant in treating NHL. Additionally, in acute myocardial infarction (AMI), Chaihulonggumulitang can accelerate the bone marrow mesenchymal stem cells- (BM-MSCs-) mediated release of inflammation and myocardial apoptosis by promoting BMSCs mobilization [10]. Eicosanoid-based therapeutic strategies such as cannabinoids contribute to improving hematopoietic transplantation by regulating CXCR4 expression and inducing HSC mobilization [11]. Accumulating evidence indicates that the intervention of TCM can enhance the mobilization effect and speed up hematopoietic function recovery and immunological reconstitution after HSC transplantation in hematological malignancies [12]. Therefore, TCM is additionally a potential effective mobilization agent for HSC after transplantation, thereby facilitating the therapeutic effect of APBSCT.

Jiedu Xiaoluo decoction (JDX) is a common recipe which is widely used in the long-term practice of Traditional Chinese Medicine. JDX also started to be used in modern medicine targeting many diseases including tumors. Combined therapy with BJD and alendronate not only synergistically fights osteoporosis by inhibiting bone resorption, but also makes breast cancer cells sensitive to endocrine treatment by promoting serum estradiol (E2) level and estrogen receptors levels in tumor tissues [13]. In animal model of transplanted primary hepatic carcinoma (PCH), JDX treatment significantly inhibits tumor growth by depressing serum vascular endothelial growth factor (VEGF) level and promoting tumor cell apoptosis [14]. These data suggest that JDX-mediated tumor inhibition is partly through regulating peripheral blood cytokines. It is confirmed that cytokines have the main effect in mobilization of peripheral blood stem cell [15]. However, whether JDX has the mobilization effect on HSC during therapeutic process of NHL using APBSCT remains unclear.

Herein, we intended to explore the synergistic effects of JDX on NHL when treated with APBSCT. We observed that the combination of APBSCT with JDX achieved the effective anti-tumor role by improving the hematopoietic and immune microenvironment, which indicated the potential role of JDX in controlling the development of NHL.

## 2. Methodology

### 2.1. Cells and Cell Culturing

Mouse lymphoma cell line A20 cells were purchased from American Type Culture Collection (ATCC). The cells were cultured in RPMI-1640 medium (SH3080901, Hyclone, USA) supplemented with 1% penicillin/streptomycin (J150019, Hyclone, USA) and 10% (v/v) fetal bovine serum (FBS, 10270-106, Gibco, USA). The cells were incubated at 37°C in a 5% CO_2_ incubator (Thermo, USA). When the cell growth reached 70–80% confluence, cells were digested and used in the next experiments.

### 2.2. Preparation of Jiedu Xiaoluo Decoction

*Astragalus* (30 g), ginseng (15 g), *Atractylodes macrocephala* (10 g), *Poria* (10 g), *Ligustrum* (15 g), dodder (15 g), wolfberry (15 g), and *Caulis spatholobi* (30 g) were soaked in cold water (7 times the volume of crude drug) for 30 minutes. The mixture was cooked for 60 min and then the decoction was filtered to remove the residue (the first decoction). After adding 3 times volume water to the residue, the mixture was boiled for 45 min again. The decoction was filtered again to collect the second decoction. Next, the decoctions were mixed and concentrated to 2 g (crude drug quantity)/ml. The decoctions were stored at 4°C for the next experiments.

### 2.3. Animals and Animal Model

Four-week-old male BALB/c-nu nude mice were purchased from Nanjing University of Traditional Chinese Medicine. All mice were housed in a specific pathogen-free room under controlled temperature and humidity. All protocols were approved by the Ethics Committee of Zhejiang Chinese Medical University and animal handling was in compliance with the National Institute of Health Guidelines for Care and Use of Animals. After a week of adjusting feeding, approximately 2 × 10^6^ (0.2 ml) A20 cells per mice were inoculated subcutaneously into the left forelimb and the right hind limb. Then, the mice were housed under a specific pathogen-free room under controlled temperature and humidity. After 3-4 weeks, the tumor size was recorded. Then, mice bearing tumors were randomly divided into three groups (*n* = 10/each group) including control group, transplanted group (tail vein injection of stem cell), and treatment group (stem cell injection plus JDX administration by gavage).

### 2.4. Peripheral Blood Mononuclear Cells Preparation and Animal Treatment

Peripheral blood mononuclear cells were isolated from the male littermates of nude mice bearing with tumors (the recipient mice). Briefly, 5 days before transplantation, 200 *μ*g/kg rhGM-CSF was started to be injected subcutaneously into mice once a day because rhGM-CSF has the function of promoting the production of hematopoietic stem cells and could influence the quality and number of hematopoietic stem cells, which were important for the following transplantation. The blood was obtained and the monocytes were separated using lymphocyte separation solution 2-3 hours after the last injection because the half-life period of rhGM-CSF is 1–3 hours. After washing twice with RPMI-1640 culture solution without serum, the cells were resuspended, stained with trypan blue to evaluate the cell viability, and adjusted to 4 × 10^7^ cells/ml. For the transplanted group, on the day of transplantation, all mice were irradiated with cobalt 60*γ*-ray for the total dose of 3.0 Gy (1.0 Gy/min). Then, 2 × 10^7^ peripheral blood mononuclear cells per mice were transplanted by intravenous injection within 4 hours after irradiation. For the JDX treatment group, 0.2 ml of JDX was given by gavage for 4 consecutive weeks (30 days). And mice in control group and transplanted group were given 0.2 ml of 0.9% normal saline by gavage every day. In the course of JDX treatment, the hair, body position, body shape, diarrhea, and death time were recorded every day. The body weight was measured every three days. From the second week after JDX administration, the longest diameter (a) and the shortest diameter (b) of the tumor mass were measured every 5 days, and the tumor volume was calculated according to the formula: tumor volume = ab^2^/2.

### 2.5. Hematopoietic Function Measurement

20 *μ*L blood samples were collected from tail vein on days 0, 3, 7, 14, 21, and 30 after the transplantation and JDX treatment, respectively. For each group, three mice were selected at every time point. Then, the peripheral blood leukocytes (WBC), red blood cells (RBC), and platelets (PLT) were detected by automatic blood analyzer. For the measurement of nucleated cells in bone marrow fluid, on the 30th day after administration or death time, the femur of the hind limb of the mice was harvested. Next, 10 ml 0.9% NaCl was used to flush out the bone marrow into the centrifuge tube. After centrifugation at 1500 rpm, the supernatant was removed and 1 ml of 10% neutral formalin was added to the cells for counting the number of bone marrow nucleated cells.

### 2.6. ELISA

The homogenate of the tumor tissue in each group was prepared and centrifuged. Then, the supernatant was detected by the corresponding ELISA kits. To evaluate the effects of JDX on the immunological environment, several cytokines were selected and their expression was measured by ELISA: (1) the selected immunity promoting cytokines were IL-2, IL-4, IL-6, IL-12, IL-17, TNF-*α*, and INF-Υ; (2) the selected immunity abating or tumor promoting proteins were IL-10, TGF-*β*, VEGF, and MMP-9. The ELISA kits for IL-2, IL-4, IL-6, IL-10, IL-12, IL-17, TNF-*α*, IFN-*γ*, TGF-*β*, and VEGF were from Hui Jia Company (China) and the ELISA kit for MMP-9 was from Thermo Fisher (USA). Two kinds of samples were detected with the ELISA detection described above: (1) the homogenate of the tumor tissue in each group was prepared and centrifuged. Then, the supernatant was detected by the corresponding ELISA kits; (2) the drug serum of mice was produced with the gavage of JDX twice a day for five consecutive times. The produced drug serum was detected by the corresponding ELISA kits. The ELISA detection procedure was following the procedure manual of each ELISA kits. Then, the absorbance in each sample was measured at the wavelength of 450 nm using a microplate reader.

### 2.7. Histological Staining

Briefly, the tumor tissues of each experimental group were fixed in 10% formaldehyde for 48 hours. After washing and dehydration, the tumor samples were embedded in paraffin. Then, 5 *μ*m thick tissue sections were prepared, dewaxed, and hydrated, and the slides were stained with hematoxylin and eosin. After mounting the sections, the image was observed under the microscope and analyzed using ImageJ software.

### 2.8. Immunohistochemistry

5 *μ*m thick tissue sections were used to perform IHC staining. In brief, the sections were roasted overnight at 37°C. After dewaxing and hydration, the antigen repair was conducted by high pressure method. Then, the slices were incubated with 3% H_2_O_2_ solution at room temperature for 10 min to block endogenous peroxidase. Next, the sections were blocked using 5% BSA for 1 h. 50 *μ*L diluted primary antibodies against CD20 (1 : 100, 60271–1, Proteintech Company, USA), Bcl-2 (1 : 200, AF6139, Affinity Company), Bcl-6 (1 : 200, bs-13606R, Bioss), and Ki67 (1 : 500, AF0198, Affinity Company) were added to each section and incubated overnight at 4°C. After washing with TBST three times, 50 *μ*L secondary antibody was added for incubation at 4°C for 20 min. The staining results were colored by adding DAB solution and photographed under a microscope. The numbers of positive cells were calculated by using ImageJ software.

### 2.9. Preparation of Drug Serum

Eight-week-old male Wistar rats (weighing 200 ± 20 g) were divided into control group (normal saline) and drug-treated group (JDX). The dosage was calculated by the following formula: volume = 2 × the dosage of clinical human × equivalent area coefficient of animal (0.018). The drug was given by gavage twice a day for five consecutive times. 2 hours after the last administration, the blood samples were collected from the inferior vena cava. After gently shaking, the blood was allowed to stand at 4°C for 4 hours. The serum was separated by centrifugation at 3000 rpm for 25 minutes. The drug serum was inactivated at 56°C for 30 minutes and stored at −20°C for the in vitro and in vivo experiments.

### 2.10. Co-Culture System and Treatment

A20 cells were divided into three groups. Control group was treated with control serum alone. For the co-culture system, approximately 1 × 10^5^ A20 cells and 1 × 10^5^ autologous hematopoietic stem cell (HSCs) suspension were plated into the lower chamber and upper chamber of Transwell apparatus, respectively. Then, 0.2 ml drug serum or control serum was added to the medium in the upper chamber. After treatment with 24 h, 48 h, and 72 h, the A20 cells were obtained for the next experiments.

### 2.11. CCK8 Assay

A20 cells in logarithmic growth stage were counted under microscope and then made into 1–5 × 10^4^ cells/ml cell suspension. 100 *μ*L cell suspension was added to the lower chamber of 96-well plate set up by eight replications. 100 *μ*L culturing medium served as blank control. After co-culturing with HSCs and treating with JDX drug serum for 24 h, 48 h, and 72 h, 10 *μ*L cell counting kit-8 (CCK-8) was added to cells and the absorbance at 450 nm wavelength was measured by a microplate reader, and the value of each plate was recorded.

### 2.12. Migration and Invasion Measurements

For the migration ability detection, the co-culturing mixture of A20 cells and HSCs was seeded at the upper chamber of Transwell apparatus; control and JDX drug serum were used to treat the co-culturing mixture cells in the upper chamber for 24 h, 48 h, and 72 h; the lower chamber was used to take photos and count the migrating A20 cells. For the invasiveness detection, the upper surface of the Transwell was coated with 50 *μ*L Matrix gel (50 mg/L) at 37°C for 4 hours; the co-culturing mixture of A20 cells and HSCs was seeded at the upper chamber of Transwell apparatus; control and JDX drug serum were used to treat the co-culturing mixture cells in the upper chamber for 24 h, 48 h, and 72 h; the lower chamber was used to take photos and count the invading A20 cells. After photographing, the number of migrations and invasions was quantified using Image J software.

### 2.13. Flow Cytometry

Briefly, the cells were collected and centrifuged at 1000–2000 rpm for 5 min. The supernatant was discarded and the cells were resuspended using 500 *μ*L binding buffer. 5 *μ*L annexin V-FITC and 5 *μ*L propidium iodide (PI) were added to the cell mixture for incubation at room temperature (20–25°C) in the dark for 15 min. Then, flow cytometry detection was performed immediately. Annexin V-FITC was green fluorescence and PI was red fluorescence.

### 2.14. Xenograft Model

A20 cells were treated with control serum, co-culture with stem cells plus control serum, and co-culture with stem cells plus JDX drug serum, respectively. Then, approximately 2 × 10^6^ (0.2 ml) A20 cells per mice were inoculated subcutaneously into the mice to establish xenograft model according to the above methods mentioned in the section of animals and animal model. The longest diameter (a) and the shortest diameter (b) of the tumor mass were measured weekly, and the tumor volume was calculated according to the following formula: tumor volume = ab^2^/2.

### 2.15. RT-PCR

Total RNA from tumor tissues was extracted by TRIzol reagents. 1 *μ*g RNA was reverse-transcribed into cDNA by using cDNA reverse transcription kit. The primer sequences are shown in Table 1. The qPCR running program was as follows: 1 cycle of 95°C for 5 min, 40 cycles of 95°C for 5 s, 60°C for 30 s and 95°C/15 s, and 1 cycle of 60°C for 30 s and 95°C for 15 s. All data were normalized to the control of GAPDH. Formula 2^−ΔΔCt^ was used to calculate the relative expression of these genes.

### 2.16. Western Blotting

Briefly, total proteins were extracted by using RIPA protein lysate (89900, Invitrogen, USA). 40 *μ*g protein was used to perform SDS-PAGE assay. Primary antibodies IL-10 (1 : 1000, Santa Cruz Company, USA), IL-12 (1 : 500, Santa Cruz Company, USA), IFN-*γ* (1 : 500, Santa Cruz Company, USA), TGF-*β* (1 : 250, Santa Cruz Company, USA), and VEGF (1 : 500, Santa Cruz Company, USA) were employed to incubate with activated membrane overnight at 4°C. GAPDH (1 : 5000) served as the internal control. Then, the bands were detected by incubation with HRP conjugate secondary antibody at room temperature for 1 h. The protein expression was conducted using an enhanced chemiluminescence reagent and the images were observed by a gel imaging analysis system.

### 2.17. Statistical Analysis

All experiments were processed at least three times. Relative quantitative analysis of protein and cell counting was processed using Image J software. All data were expressed as mean ± standard deviation. Data analysis was determined by two-tailed Student's *t*-test and two-way ANOVA by using GraphPad prism software (GraphPad Software, Inc.). *P* < 0.05 was considered to indicate statistically significant differences.

## 3. Results

### 3.1. JDX Management Promotes Hematopoietic Recovery after Autologous Peripheral Blood Stem Cell Transplantation (APBSCT)

**Figure 1:**
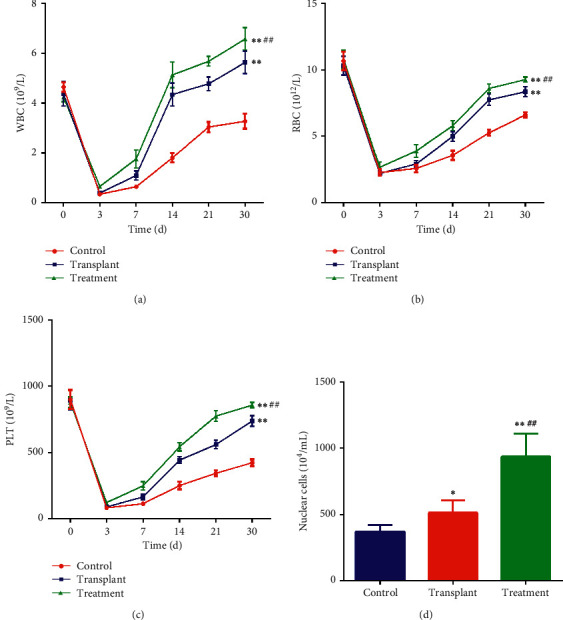
Effect of JDX on hematopoietic function after APBSCT in mice bearing lymphoma cells. The numbers of white blood cells (WBC) (a), red blood cells (RBC) (b), and platelets (PLT) (c) in peripheral blood as analyzed by automatic blood analyzer in control, transplant, and treatment group. (d) The number of nucleated cells as calculated in bone marrow fluid of the above three groups. The control group is orally administrated with 0.2 ml 0.9% normal saline daily (*n* = 10). The transplant group was challenged by 0.2 ml 0.9% normal saline daily after APBSCT (*n* = 10). The treatment group was challenged with 0.2 ml Jiedu Xiaoluo decoction after APBSCT (*n* = 10). ^∗^Control group vs transplant group and treatment group. ^#^Treatment group vs transplant group. ^∗^*P* < 0.05; ^∗∗^ or ^##^*P* < 0.01.

The treatment effect of APBSCT is always restricted by delayed hematopoietic recovery during treating NHL. To address this issue, we employed JDX as a regimen adjuvant of APBSCT. As shown in Figure 1, in mice bearing tumors (A20 cells), the numbers of WBC, RBC, and PLT decreased significantly after 3 d of the whole-body irradiation with cobalt 60 (*P* < 0.01). However, from day 7 after APBSCT, as the time passed, the number of RBC, WBC, and PLT in the peripheral blood of the transplant group robustly increased compared to control group (*P* < 0.01) (Figures 1(a)–1(c), the blue vs the red). When compared with those in the control group, the numbers of RBC, WBC, and PLT showed more in the JDX treatment group than those in the transplant group alone with the increase in time (*P* < 0.01) (Figures 1(a)–1(c), the green vs the blue). Additionally, stem cell transplantation also enhanced the number of nucleated cells in bone marrow fluid compared to control group (*P* < 0.05) (Figure 1(d)). Of note, the combination of JDX and APBSCT presented the largest number of nucleated cells when compared to control and transplantation group (*P* < 0.01) (Figure 1(d)). In summary, the results demonstrated that JDX could accelerate the recovery and mobilization of HSC in the process of NHL treatment with APBSCT.

### 3.2. Combination of APBSCT with JDX Inhibits Tumor Growth and Ameliorates Tumor Microenvironment (TME)

**Figure 2:**
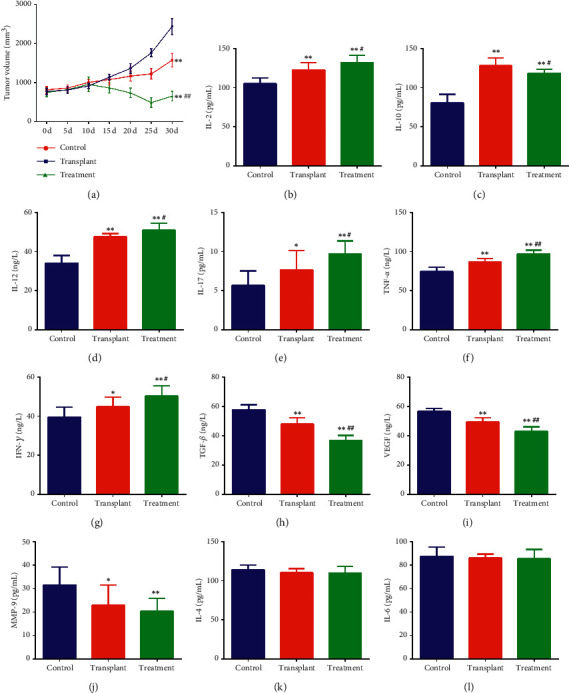
The comparison of the tumorigenesis indexes and immune microenvironmental factors in different groups. (a) The tumor volume curve of control, transplant, and treatment group. The tumor volume was calculated every five days after transplantation or JDX administration. Levels of IL-2 (b), IL-10 (c), IL-12 (d), IL-17 (e), TNF-*α* (f), INF-*γ* (g), TGF-*β* (h), VEGF (i), MMP-9 (j), IL-4 (k), and IL-6 (l) in the above three groups as measured by their corresponding ELISA kits. ^∗^Control group vs transplant group and treatment group. ^#^Treatment group vs transplant group. ^∗^ or ^#^*P* < 0.05; ^∗∗^ or ^##^*P* < 0.01.

Next, we evaluated the effect of the combination treatment on tumor growth of NHL. The results showed that the approximant tumor volume sizes of BALB/c-nu mice were significantly reduced from day 15 to day 30 after APBSCT compared to control group (Figure 2(a), the blue vs the red). However, in comparison with control group, the combination of APBSCT and JDX notably inhibited tumor growth from day 10 after stem cell transplantation, showing stronger inhibition effect than APBSCT alone (Figure 2(a), the green vs the blue). ELISA assay was performed to explore the changes of TME in these groups. The levels of IL-2, IL-10, IL-12, IL-17, TNF-*α*, and IFN-*γ* in tumor tissues were obviously increased in the transplant group compared to the control mice (Figures 2(b)–2(g)). The addition of JDX after APBSCT further enhanced the secretion of these cytokines except IL-10, which was also elevated compared to control group (Figures 2(b)–2(g)). Besides, the content of TGF-*β*, VEGF, and MMP-9 decreased significantly in the transplant group compared with control tumor, whose expression showed lower levels in the combination group than that in the transplant group alone (Figures 2(h)–2(j)). However, the number of IL-4 and IL-6 had no significant difference among the three groups (Figures 2(k)–2(l)). Therefore, JDX might be an effective immunoregulator in TME, thereby promoting the anti-tumor effect of APBSCT during NHL treatment.

### 3.3. Combination of APBSCT with JDX Remits Histopathology and Promoted Cell Apoptosis in Mice Bearing NHL

**Figure 3:**
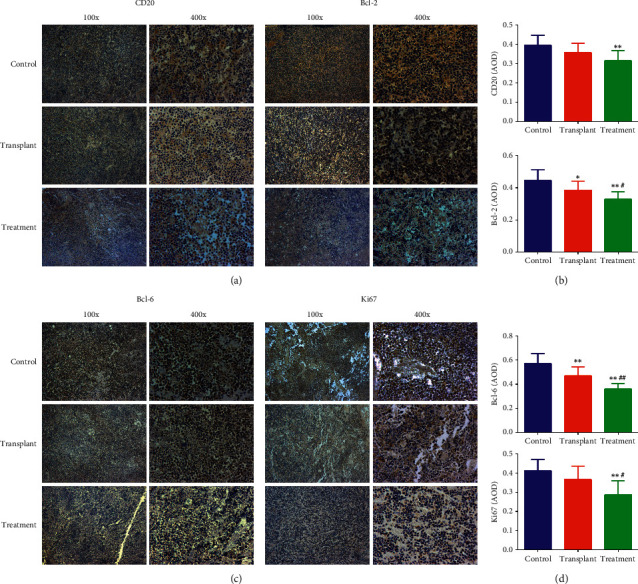
Immunohistological detection for the expression and distribution of CD20, Bcl-2, Bcl-6, and Ki67 in tumor area of mice in each group. The expression of CD20 (a) and Bcl-2 (b) as determined by immunohistochemical staining in tumor tissue of each group. (c) The area of density (AOD) of CD20- and Bcl-2-positive cells in the three groups. The expression of Bcl-6 (d) and Ki67 (e) as determined by immunohistochemical staining in tumor tissue of each group. (f) The area of density (AOD) of Bcl-6- and Ki67-positive cells in the three groups. 100x or 400x indicates the magnification. ^∗^Control group vs transplant group and treatment group. ^#^Treatment group vs transplant group. ^∗^ or ^#^*P* < 0.05; ^∗∗^ or ^##^*P* < 0.01.

In comparison to the control tumor, the number of the tumor cells with hyperchromatic nuclei and scant cytoplasm was less in the transplantation group. And cells with nuclear fission showed more in control group than those in transplantation group, while, in the treatment group, the tumor cells were even less. There were many necrotic areas in stem cell transplantation group compared to control tumor. Of note, the APBSCT-mediated remission of histopathology in tumor tissues was enlarged when JDX was added (Figure S1). We next assessed the expression and distribution of CD20, Bcl-2, Bcl-6, and Ki67, which were the key proliferation- and anti-apoptosis-related proteins. And the results showed that CD20 and Bcl-2 were mainly expressed in cytoplasm, and Bcl-6 and Ki67 were highly expressed in cytoplasm and nucleus in control group (Figures 3(a)-3(b) and 3(e)-3(f)). The expression of Bcl-2 and Bcl-6 in the transplant group was notably downregulated compared to control tumor (Figures 3(b), 3(d), 3(c), and 3(f)). But the expression of CD20, Bcl-2, Bcl-6, and Ki67 in the JDX treatment group was further decreased compared with control group (Figures 3(a)–3(e)). In comparison to transplantation group, the levels of Bcl-2, Bcl-6, and Ki67 were lower in the combination group (Figures 3(b)–3(f)). There also was a moderate decrease in the numbers of CD20-positive cells when compared the transplantation group with the JDX treatment group, showing no significant difference between them (Figures 3(a) and 3(c)). The above results demonstrate that combination of APBSCT with JDX impeded tumor growth of NHL partly through inhibiting the expression of proliferation- and anti-apoptosis factors.

### 3.4. Serum Containing JDX Elevates HSCs-Evoked Inhibition of Growth, Migration, and Invasion in A20 Cells

**Figure 4:**
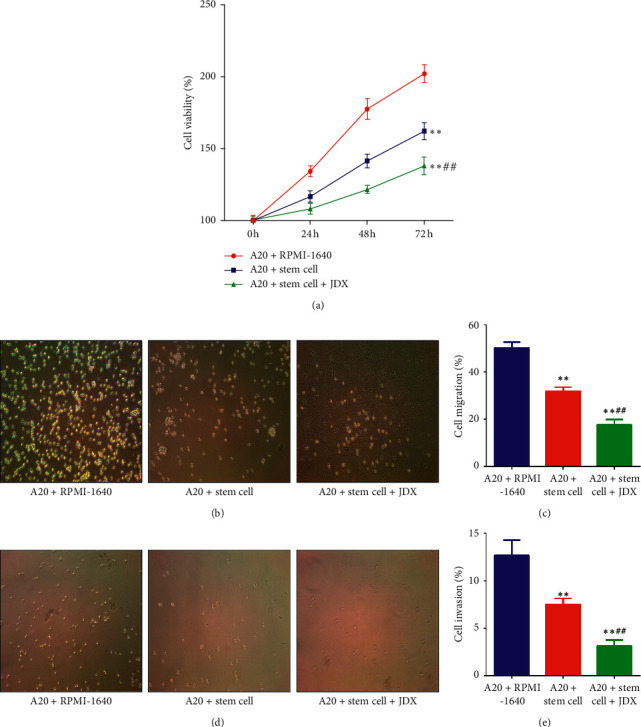
Effect of drug serum containing JDX on the proliferation, migration, and invasiveness of A20 cells. (a) The cell viability as measured by CCK8 assay in control, co-culture with HSCs, and drug serum treated group. (b) Measurement of cell migration as detected by Transwell assay in the above three groups. (c) The numbers of migration cells in the three groups. (d) Detection of cell invasion as detected by Transwell assay in the above three groups. (e) The numbers of invasion cells in the three groups. The control group was treated with control serum. The co-culture with HSCs group was treated with control serum in the co-culture system of A20 cells and HSCs. The drug serum group was treated with serum containing JDX in the co-culture system of A20 cells and HSCs. ^∗^Control group vs co-culture with HSCs group and drug serum group. ^#^Drug serum group vs co-culture with HSCs group. ^∗^ or ^#^*P* < 0.05; ^∗∗^ or ^##^*P* < 0.01.

To further prove the role of serum containing JDX in APBSCT-mediated tumor suppression, the co-culture system of A20 cells and HSCs was established. As shown in Figure 4(a), compared with the control cells, the cell viability of A20 cells was significantly inhibited (*P* < 0.01) when co-cultured with HSCs (Figure 4(a), the blue vs the red). Once the serum containing JDX was added, the HSCs-mediated inhibitory effect on cell proliferation was magnified (Figure 4(a), the green vs the blue). Combined with Transwell assay, we observed that the numbers of migration and invasion A20 cells were decreased significantly after the intervention of HSCs compared to control cells, which was further depressed when cells were treated with the combination of HSCs and serum containing JDX (Figures 4(b)–4(e)). The above results inferred that serum containing JDX also has the acceleration role on HSCs-evoked anti-tumor effect in in vitro A20 cells.

### 3.5. Serum Containing JDX Promotes HSCs-Mediated Apoptosis of A20 Cells

**Figure 5:**
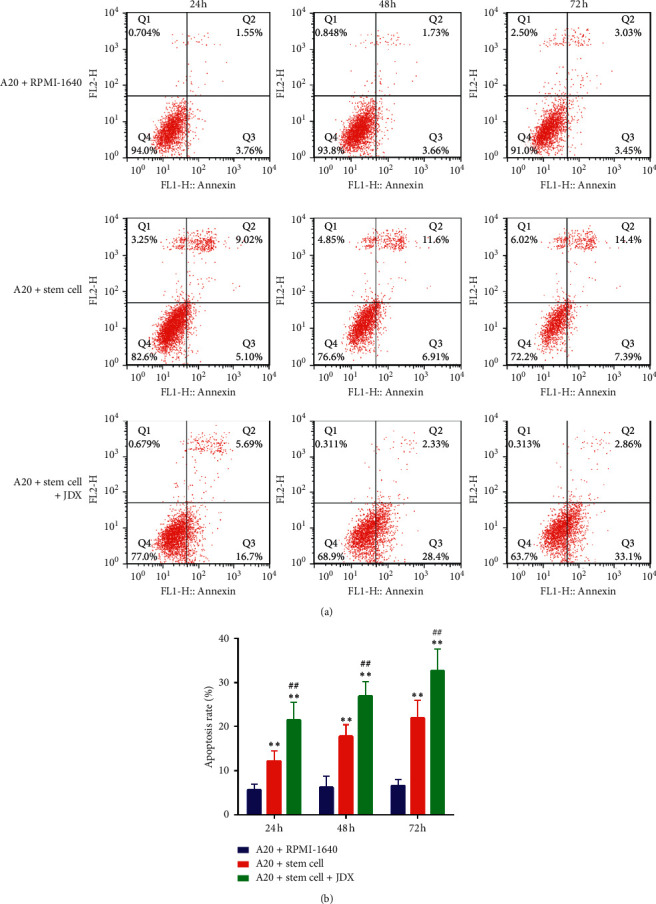
Effect of drug serum containing JDX on HSCs-mediated cell apoptosis of A20 cells. (a) The representative image of apoptosis cells in control, co-culture with HSCs, and drug serum treated group as measured by flow cytometry. (b) The statistical analyses of the apoptotic cell rate in the above three groups.

Combined with flow cytometry, we also monitored the effect of drug serum containing JDX on apoptotic events of A20 cells. In the co-culture system, HSCs presence obviously increased the number of apoptotic A20 cells over time, increasing by three times, four times, and five times at 24 h, 48 h, and 72 h compared to control cells (RPMI-1640), respectively (Figures 5(a)-5(b)). Additionally, under the co-treatment of HSCs and drug serum containing JDX, the results showed that the apoptosis rate of A20 cells increased by 5 times, 6 times, and 7 times at 24 h, 48 h, and 72 h compared with the control group, respectively (Figures 5(a)-5(b)). Remarkably, the combination-evoked pro-apoptosis effect was larger than that of the HSCs alone, showing 1.5-fold increase in the presence of JDX compared to HSCs alone (Figures 5(a)-5(b)). Thus, the data indicated that serum containing JDX has synergistic effect in promotion of A20 cell apoptosis induced by HSCs co-culture.

### 3.6. Serum Containing JDX Boosts HSCs-Mediated Immunoregulation of A20 Cells

**Figure 6:**
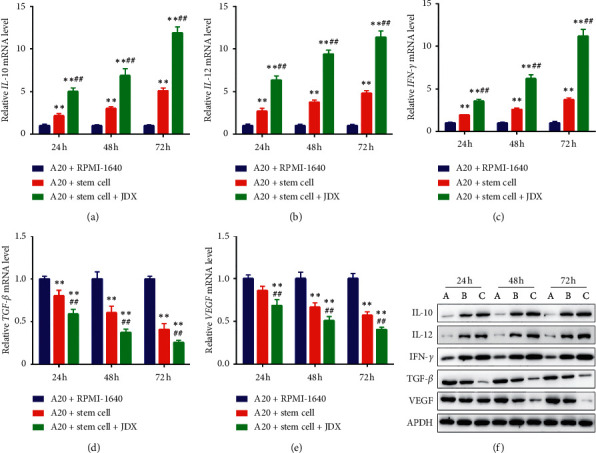
The mRNA and protein levels of inflammatory cytokines in A20 cells. (a) Transcriptional level of IL-10 (a), IL-12 (b), IFN-*γ* (c), TGF-*β* (d), and VEGF (e) as determined by qRT-PCR in control, co-culture with HSCs, and drug serum treated group. Cells were treated at 24 h, 48 h, and 72 h, respectively. (f) The protein levels of IL-10, IL-12, IFN-*γ*, TGF-*β*, and VEGF as detected by western blotting. GAPDH served as the internal control. ^∗^Control group vs co-culture with HSCs group and drug serum group. ^#^Drug serum group vs co-culture with HSCs group. ^∗^ or ^#^*P* < 0.05; ^∗∗^ or ^##^*P* < 0.01.

For the immunoregulatory effect of JDX on NHL tumor in vivo, we also discovered that the transcriptional levels of IL-10, IL-12, and IFN-*γ* in A20 cells were significantly increased when A20 cells were co-cultured with HSCs, which were further elevated and showed the highest expression in the combination treatment of HSCs and drug serum compared to control and HSCs-treatment alone (Figures 6(a)–6(c)). The mRNA expressions of TGF-*β* and VEGF were significantly reduced for HSCs alone and the combination group compared to control cells (Figures 6(d)-6(e)). And the above effects were more significant in the combination group than that in HSCs alone group (Figures 6(d)-6(e)). Additionally, the protein levels of IL-10, IL-12, and IFN-*γ* were obviously enhanced, while the protein levels of TGF-*β* and VEGF were notably decreased in HSCs co-culture group and medication-combination group, showing a stronger effect in the combination group than that in HSCs alone group (Figures 6(f) and Figures S2(a)–S2(e)). In the xenograft model, drug serum containing JDX also played positive effects on APBSCT-induced tumor suppression. As shown in Figure S3, tumor volume and tumor weight were robustly reduced under the pre-stimulation of HSCs compared to those in control group; that reduction was more remarkable in the combination group (Figures S3(a)-S3(b)). The above results suggested the following: (1) for the co-culturing system of A20 cells with HSCs, the combination treatment could significantly modulate the expression of inflammatory cytokines, thereby inhibiting tumor characteristics of A20 cells; (2) for the xenograft model from A20 cells, the combination treatment could significantly reduce the tumor progression. Of note, drug serum containing JDX might act as the effective conditioning regimen to promote HSCs mobilization by immunoregulation, resulting in the tumor cell growth inhibition of A20 cells and the suppression of tumor progression.

### 3.7. The Gavage of JDX Increased the Expression of Immunity Promoting Factors and Decreased the Expression of Immunity Abating Factors

**Figure 7:**
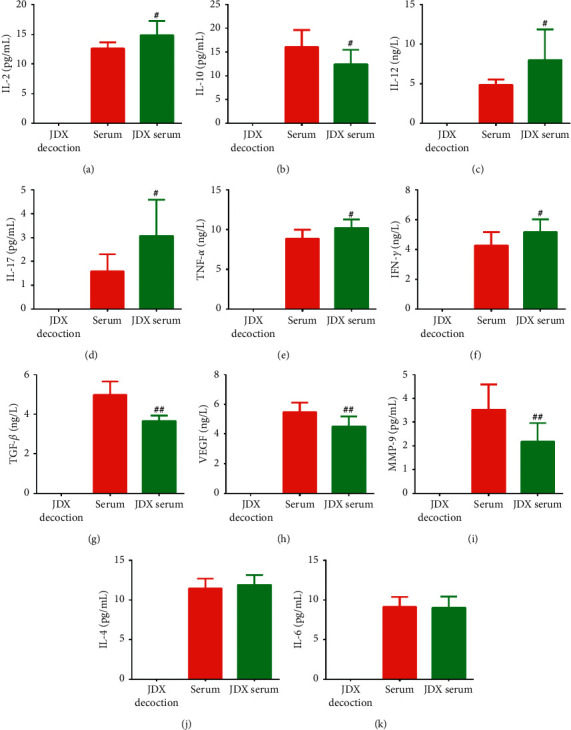
The ELISA results for the cytokines or proteins in the drug serum, JDK decoction, and the control serum. (a) IL-2; (b) IL-10; (c) IL-12; (d) IL-17; (e) TNF-*α*; (f) IFN-*γ*; (g) TGF-*β*; (h) VEGF; (i) MMP-9; (j) IL-4; (k) IL-6. ^#^, *P* < 0.05; ^##^, *P* < 0.01, compared with the control serum.

In order to confirm the effects of JDX on the immunological environment, the mouse drug serum produced by the gavage of JDX was detected for the expression of several immunity affecting factors including (1) the immunity promoting cytokines such as IL-2, IL-4, IL-6, IL-12, IL-17, TNF-*α*, and INF-Υ and (2) the immunity abating or tumor promoting proteins such as IL-10, TGF-*β*, VEGF, and MMP-9. The results showed that IL-2, IL-12, IL-17, TNF-*α*, and INF-Υ, which were immunity promoting cytokines, were significantly upregulated in drug serum, while IL-10, TGF-*β*, VEGF, and MMP-9, which were immunity abating or tumor promoting proteins, were significantly downregulated in drug serum (Figure 7). In summary, JDX promoted the immunity of the mouse receiving the JDX gavage and the effect of JDX drug serum on A20 was considered at least partially from the expression alteration of these cytokines or proteins.

## 4. Discussion

NHL comprises a wide spectrum of lymphoid neoplasms derived from T cells, natural killer cells, or B cells that affect approximately 1.5 million people worldwide. Among them, B-cell lymphomas (BCLs) account for more than 85% of cases of NHL [16]. Anti-CD20 immunotherapy combined with chemotherapy agents was considered as the first-line option [17]. Though 3-year progression-free survival (PFS) of NHL patients was obviously enhanced, many patients relapse [18]. Accumulating evidence reveals that APBSCT is an effective treatment for certain patients with refractory or recurrent NHL [19]. In the present study, JDX or serum containing JDX management facilitated APBSCT-mediated tumor inhibition of A20 cells by HSCs mobilization and immunoregulation. Thus, JDX or serum containing JDX has the potential to evolve into a conditioning regimen of APBSCT during treating recurrent NHL.

Early absolute lymphocyte count (ALC) recovery has been proved as a consistent predictor of improved survival after APBSCT [20]. Oral calcitriol can significantly increase the short-term and long-term hematopoietic recovery after APBSCT by enhancing the numbers of white blood cells (WBC) and platelets (PLT) in Hodgkin's lymphoma (HL), NHL, and multiple myeloma (MM) patients [21]. Dose-adjusted cyclophosphamide, doxorubicin, vincristine, and prednisolone (CHOP) chemotherapy can effectively mobilize APBSCs and elevate the complete response rate in NHL patients, facilitating the treatment of NHL [22]. In our study, after APBSCT, the hematopoietic recovery was apparently presented along with the increase of WBC, RBC, and PLT amounts. That indicated that autologous HSCs transplantation was successful and effective. Additionally, it is confirmed that panax notoginsenosides (TCM) improves left ventricular function after acute myocardial infarction through promoting the mobilization of bone marrow-derived stem cell [23]. Icaritin contributes to the myelosuppression by modifying bone marrow hematopoietic microenvironment and promoting the proliferation and differentiation of HSCs [24]. The above research implies that the use of TCM can improve the mobilization viability of HSCs and hematopoietic recovery. Herein, the administration of JDX effectively magnified the APBSCT-mediated improvement of hematopoietic function in mice bearing NHL tumors, which suggested that JDX might be a key determinant for promoting mobilization of HSCs after APBSCT.

Immune reconstitution in TME is another key event after APBSCT [25]. The inability of the cytokine in TME can induce rearrangements of adhesive interactions within the hematopoietic tissue, thereby increasing the metastasizing potential of hematological malignancies [26]. Immune reconstitution including the increase of IL-2, IL-4, IL-10, TNF-*α*, and IFN-*γ* is observed in intermediate grade NHL patients after high-dose chemotherapy (HDT) and stem cell transplantation (SCT) [27]. Recombinant human IL-12 remits patients with relapsed NHL by increasing the number of circulating CD8+ cells [28]. The T-helper cell 1/T-helper cell 2 (Th1/Th2), that is responsible for the secretion of IL-2, IFN-*γ*, TNF-*α*, and the secretion of IL-4, IL-5, IL-10, and IL-13, respectively, is decreased initially, and increased after the cessation of G-CSF, following APBSCT, leading to an increase in lymphocytes in NHL patients [29]. In hematological malignancies, the use of biosimilar G-CSF has successfully mobilized the autologously transplanted or allogenetically transplanted stem cells, promoting the engraftment of the transplants [30]. Therefore, immunological reconstitution after APBSCT is crucial to treat NHL. The present data indicated that JDX or serum containing JDX management contributed to the tumor growth inhibition effect and reconstitution of tumor inflammatory microenvironment by enhancing the secretion and expression of IL-2, IL-10, IL-12, IL-17, TNF-*α*, and IFN-*γ* and inhibiting the levels of TGF-*β*, VEGF, and MMP-9 in mice bearing lymphoma tumor after APBSCT. MMP-9 and VEGF are angiogenic factors and have prognostic value of angiogenesis in childhood NHL [31]. TGF-*β* mediates effector memory T-cell exhaustion and T cell differentiation in B-cell NHL, thereby exerting an immunosuppression effect [32, 33]. In summary, the amplified action of JDX or JDX drug serum addition after APBSCT on tumor growth was potentially through regulating the balance of Th1/Th2 cells and downstream the T cell activity, resulting in the hindrance of angiogenesis in TME of B-cell NHL.

Currently, JDX has been exploited to treat several diseases. BJD relieves diabetic osteoporosis by activating the Wnt signaling while inhibiting NF-*κ*B signaling pathway [34]. In chemically damaged mice, JDX treatment relieves the injury symptom by upregulating the expression of Wnt3a in bone marrow mononuclear cells [35]. Jiedu Xiaoluo Qingchang Huashi Recipe (JBQHR) could promote proliferation of bone marrow mesenchymal stem cells by regulating ERK/CREB signaling pathway [36]. BJD administration prolongs the survival of liver cancer patients by inducing apoptosis along with the regulation of PI3K, Akt, p53, CASP3, and Bcl-xL/BAD ratio [37]. In our study, JDX addition promoted the inhibition effect of APBSCT-mediated alteration of proliferation- (Ki67) and anti-apoptosis-associated molecules (Bcl-2/6). Besides, drug serum containing JDX also inhibited cell viability, migration, and invasion of B-cell lymphoma in vitro possibly by upregulating HSCs-evoked apoptotic events. Bcl-2 inhibitor with bevacizumab is currently an effectively therapeutic strategy to treat B-cell NHL [38]. Thus, both JDX and JDX drug serum exerted tumor suppression role by promoting the apoptosis process of A20 cells after APBSCT or co-culture with HSCs. Whether the regulation of cell migration or invasion by JDX or JDX drug serum was through regulating ERK/CREB signaling transduction remained unclear and needed to be studied further.

## 5. Conclusion

Collectively, in this study, we found that JDX administration could strengthen the effects of APBSCT to treat B-cell NHL. Mechanistically, JDX treatment contributes to the hematopoietic recovery, and downstream the regulation of inflammatory TME by maintaining the balance of Th1/Th2, thereby significantly blocking the development of B-cell NHL. Besides, drug serum containing JDX also exerted inhibition effect on the growth and metastasis of B-cell lymphoma by promoting cell apoptosis. In summary, our data provided an improvement strategy for APBSCT on treating B-cell NHL. Possibly, the combination of APBSCT with the conditioning regimen of JDX helped improve therapeutic effect on recurrent NHL.

## Figures and Tables

**Table 1:** The primer sequences of qRT-PCR.

Gene	Forward primer	Reverse primer
IL-10	CATACTGCTAACCGACTCCT	AATGCTCCTTGATTTCTGG
IL-12	ACTTGAGTTGCTACAGGGTTTCC	GACAAAGCAGCACCACAGGAC
IFN-*γ*	AGCAACAACATAAGCGTCAT	CCTCAAACTTGGCAATACTCA
TGF-b	AAGTCGGATGTGGAAATGGA	TTCTGGTTGTCGCAAGTGGA
VEGF	GCTACTGCCGTCCGATTGAG	GCTGGCTTTGGTGAGGTTTG
GAPDH	GCAGTAAACAGTCCATCTACAA	CTCTCCTTCATCCACCCT

## Data Availability

The data used to support the findings of this study are available from the corresponding author upon request.

## Abbreviations

APBSCT: Autologous peripheral blood stem cell transplantation.

## Supplementary Materials

Supplementary Materials
*Figure S1*. Comparison of changes in tumor histology. The tumor histology was determined by hematoxylin and eosin staining. Tumor tissues were harvested from control, transplant, and treatment group. The control group is orally administrated with 0.2 ml 0.9% normal saline daily (*n* = 10). The transplant group was challenged by 0.2 ml 0.9% normal saline daily after APBSCT (*n* = 10). The treatment group was challenged with 0.2 ml Jiedu Xiaoluo decoction after APBSCT (*n* = 10). Magnification: left (100x) and right (400x). *Figure S2*. Semiquantitative analysis of inflammation-related protein in A20 cells. In the co-culture system, cells were treated with control serum and drug serum for 24 h, 48 h, and 72 h. Semiquantitative analysis of IL-10 (a), IL-12 (b), VEGF (c), IFN-*γ* (d), and TGF-*β* (e) as analyzed by using ImageJ software. *Figure S3*. Establishment of xenograft model of A20 cells. (a) Tumor growth curve of mice bearing A20 cells treated with control serum and drug serum. (b) Statistic data of tumor weight in the above three groups. ^∗^Control group vs co-culture with HSCs group and drug serum group. ^#^Drug serum group vs co-culture with HSCs group. ^∗^ or ^#^*P* < 0.05; ^∗∗^ or ^##^*P* < 0.01.

## References

[1] Ansell S. M. (2015). Non-Hodgkin lymphoma: diagnosis and treatment. *Mayo Clinic Proceedings*.

[2] Jiang M., Bennani N. N., Feldman A. L. (2017). Lymphoma classification update: B-cell non-Hodgkin lymphomas. *Expert Review of Hematology*.

[3] Sekiguchi Y., Wakabayashi M., Takizawa H. (2019). Retrospective analysis of 20 patients with DLBCL who received MCVAC followed by autologous peripheral blood stem cell transplantation. *Gan to Kagaku Ryoho. Cancer & Chemotherapy*.

[4] Porrata L. F., Inwards D. J., Ansell S. M. (2008). Early lymphocyte recovery predicts superior survival after autologous stem cell transplantation in non-Hodgkin lymphoma: a prospective study. *Biology of Blood and Marrow Transplantation*.

[5] You B., Salles G., Bachy E. (2015). Etoposide pharmacokinetics impact the outcomes of lymphoma patients treated with BEAM regimen and ASCT: a multicenter study of the lymphoma study association (LYSA). *Cancer Chemotherapy and Pharmacology*.

[6] Wang X., Wang N., Cheung F., Lao L., Li C., Feng Y. (2015). Chinese medicines for prevention and treatment of human hepatocellular carcinoma: current progress on pharmacological actions and mechanisms. *Journal of Integrative Medicine*.

[7] Guo Q. J., Lin J. Y., Liu R. (2015). Review on the applications and molecular mechanisms of Xihuang pill in tumor treatment. *Evidence-Based Complementary and Alternative Medicine*.

[8] Xie Y., Li B., Bu W. (2018). Dihydrocelastrol exerts potent antitumor activity in mantle cell lymphoma cells via dual inhibition of mTORC1 and mTORC2. *International Journal of Oncology*.

[9] Lin W.-f., Lu J.-y., Cheng B.-b., Ling C.-q. (2017). Progress in research on the effects of traditional Chinese medicine on the tumor microenvironment. *Journal of Integrative Medicine*.

[10] Wang C., Du H. S., Hou J. Q. (2018). Chaihulonggumulitang shows psycho-cardiology therapeutic effects on acute myocardial infarction by enhancing bone marrow mesenchymal stem cells mobilization. *Scientific Reports*.

[11] Hoggatt J., Pelus L. M. (2010). Eicosanoid regulation of hematopoiesis and hematopoietic stem and progenitor trafficking. *Leukemia*.

[12] Sun C. Y., Wang M. S., Yang S. L. (2008). Progress on application of traditional Chinese medicine in hemopoietic stem cell transplantation. *Zhongguo Zhong Xi Yi Jie He Za Zhi*.

[13] Huang X.-H., Liang R.-H., Su L., Guo W., Wang C.-J. (2017). Mechanism of Bushen Jianpi decoction in preventing and treating osteoporosis caused by aromatase inhibitors in breast cancer treatment. *Cancer Biomarkers*.

[14] Zhong Y., Luo C. L., An-Jun Z. (2011). Effect of bushen jianpi decoction and its disassemble recipes on tumor growth in mice with transplanted primary hepatic carcinoma. *Zhongguo Zhong Xi Yi Jie He Za Zhi*.

[15] Melve G. K., Ersvaer E., Roy K. (2018). The healthy donor profile of immunoregulatory soluble mediators is altered by stem cell mobilization and apheresis. *Cytotherapy*.

[16] Chaudhari K., Rizvi S., Syed B. A. (2019). Non-Hodgkin lymphoma therapy landscape. *Nature Reviews Drug Discovery*.

[17] Shanehbandi D., Majidi J., Kazemi T., Baradaran B., Aghebati-Maleki L. (2017). CD20-based immunotherapy of B-cell derived hematologic malignancies. *Current Cancer Drug Targets*.

[18] Ying Z., Mi L., Wang L. (2017). Prognostic value of pre- and post-transplantation ^18^F-fluorodeoxyglucose positron emission tomography results in non-Hodgkin lymphoma patients receiving autologous stem cell transplantation. *Chinese Journal of Cancer Research*.

[19] Won S. C., Han J. W., Kwon S. Y. (2006). Autologous peripheral blood stem cell transplantation in children with non-Hodgkin’s lymphoma: a report from the Korean society of pediatric hematology-oncology. *Annals of Hematology*.

[20] Porrata L. F., Inwards D. J., Micallef I. N., Ansell S. M., Geyer S. M., Markovic S. N. (2002). Early lymphocyte recovery post-autologous haematopoietic stem cell transplantation is associated with better survival in Hodgkin’s disease. *British Journal of Haematology*.

[21] Raoufinejad K., Shamshiri A. R., Pezeshki S. (2019). Oral calcitriol in hematopoietic recovery and survival after autologous stem cell transplantation: a randomized clinical trial. *DARU Journal of Pharmaceutical Sciences*.

[22] Shi Y. K., Zhou P., Han X. H. (2015). Autologous peripheral blood stem cell mobilization following dose-adjusted cyclophosphamide, doxorubicin, vincristine, and prednisolone chemotherapy alone or in combination with rituximab in treating high-risk non-Hodgkin’s lymphoma. *Chinese Journal of Cancer*.

[23] ZhouJ J., Wu R., Wang L. (2018). Panax notoginsenosides protects left ventricular function after acute myocardial infarction by enhancing bone marrow-derived stem cell mobilization. *Xi Bao Yu Fen Zi Mian Yi Xue Za Zhi*.

[24] Sun C., Yang J., Pan L. (2018). Improvement of icaritin on hematopoietic function in cyclophosphamide-induced myelosuppression mice. *Immunopharmacology and Immunotoxicology*.

[25] Binder M., Rajkumar S. V., Lacy M. Q. (2019). Peripheral blood biomarkers of early immune reconstitution in newly diagnosed multiple myeloma. *American Journal of Hematology*.

[26] Santucci M. A., Lemoli R. M., Tura S. (1997). Peripheral blood mobilization of hematopoietic stem cells: cytokine-mediated regulation of adhesive interactions within the hematopoietic microenvironment. *Acta Haematologica*.

[27] Singh R. K., Varney M. L., Leutzinger C. (2007). Immune reconstitution after autologous hematopoietic transplantation with Lin−, CD34+, Thy-1lo selected or intact stem cell products. *International Immunopharmacology*.

[28] Younes A., Pro B., Robertson M. J. (2004). Phase II clinical trial of interleukin-12 in patients with relapsed and refractory non-Hodgkin’s lymphoma and Hodgkin’s disease. *Clinical Cancer Research*.

[29] Mukai M., Bohgaki T., Kondo M., Notoya A., Kohno M. (2001). Changes in the T-helper cell 1/T-helper cell 2 balance of peripheral T-helper cells after autologous peripheral blood stem cell transplantation for non-Hodgkin’s lymphoma. *Annals of Hematology*.

[30] Schmitt M., Hoffmann J.-M., Lorenz K., Publicover A., Schmitt A., Nagler A. (2016). Mobilization of autologous and allogeneic peripheral blood stem cells for transplantation in haematological malignancies using biosimilar G-CSF. *Vox Sanguinis*.

[31] Citak E. C., Oguz A., Karadeniz C., Akyurek N. (2008). Role of gelatinases (MMP-2 and MMP-9), TIMP-1, vascular endothelial growth factor (VEGF), and microvessel density on the clinicopathological behavior of childhood non-Hodgkin lymphoma. *Pediatric Hematology and Oncology*.

[32] Yang Z.-Z., Grote D. M., Xiu B. (2014). TGF-*β* upregulates CD70 expression and induces exhaustion of effector memory T cells in B-cell non-Hodgkin’s lymphoma. *Leukemia*.

[33] Yang Z. Z., Grote D. M., Ziesmer S. C. (2013). Soluble and membrane-bound TGF-beta-mediated regulation of intratumoral T cell differentiation and function in B-cell non-hodgkin lymphoma. *Plos One*.

[34] Zhang Y., Liu M., Li H. (2017). Traditional Chinese medicine Bushen-Jianpi-Huoxue decoction prevents diabetic osteoporosis in rats via Wnt and nuclear factor-kappa B signaling pathways. *International Journal of Rheumatic Diseases*.

[35] He D. C., Xiao J. J., Zhang Y., Lin H., Ding X. J., Tu Y. (2014). Effect of the Jianpi Bushen Prescription on the expression of SHP-1, Wnt3a, and AP-1 proteins in chemically damaged mice. *Genetics and Molecular Research*.

[36] Zhu L., Shen H., Liu L. (2016). Effect of Jiedu Xiaoluo Qingchang Huashi recipe on proliferation of bone marrow mesenchymal stem cells. *Zhongguo Zhong Xi Yi Jie He Za Zhi*.

[37] Wu R., Li X. Y., Wang W. H. (2019). Network pharmacology-based study on the mechanism of bushen-jianpi decoction in liver cancer treatment. *Evidence-based Complementary and Alternative Medicine*.

[38] Wang L., Peng S., Sun W., Liu X. (2019). Bevacizumab synergises with the BCL 2 inhibitor venetoclax to effectively treat B‐cell non‐Hodgkin’s lymphoma. *European Journal of Haematology*.

