# Supplementary material for: The Combination of Jiedu Xiaoluo Decoction with Autologous Peripheral Blood Stem Cell Transplantation (APBSCT) Accelerates Disease Remission of Non-Hodgkin Lymphoma
Source: Evid Based Complement Alternat Med. 2021 Jan 8;2021:2745705. doi: 10.1155/2021/2745705 (PMC7810534; doi:10.1155/2021/2745705)
Supplement: Supplementary Materials — Figure S1. Comparison of changes in tumor histology. The tumor histology was determined by hematoxylin and eosin staining. Tumor tissues were harvested from control, transplant, and treatment group. The control group is orally administrated with 0.2 ml 0.9% normal saline daily (n = 10). The transplant group was challenged by 0.2 ml 0.9% normal saline daily after APBSCT (n = 10). The treatment group was challenged with 0.2 ml Jiedu Xiaoluo decoction after APBSCT (n = 10). Magnification: left (100x) and right (400x). Figure S2. Semiquantitative analysis of inflammation-related protein in A20 cells. In the co-culture system, cells were treated with control serum and drug serum for 24 h, 48 h, and 72 h. Semiquantitative analysis of IL-10 (a), IL-12 (b), VEGF (c), IFN-γ (d), and TGF-β (e) as analyzed by using ImageJ software. Figure S3. Establishment of xenograft model of A20 cells. (a) Tumor growth curve of mice bearing A20 cells treated with control serum and drug serum. (b) Statistic data of tumor weight in the above three groups. ∗Control group vs co-culture with HSCs group and drug serum group. #Drug serum group vs co-culture with HSCs group. ∗ or #P < 0.05; ∗∗ or ##P < 0.01. [file 2745705.f1.doc]

# Supplementary Materials

Supplementary Figure S1:


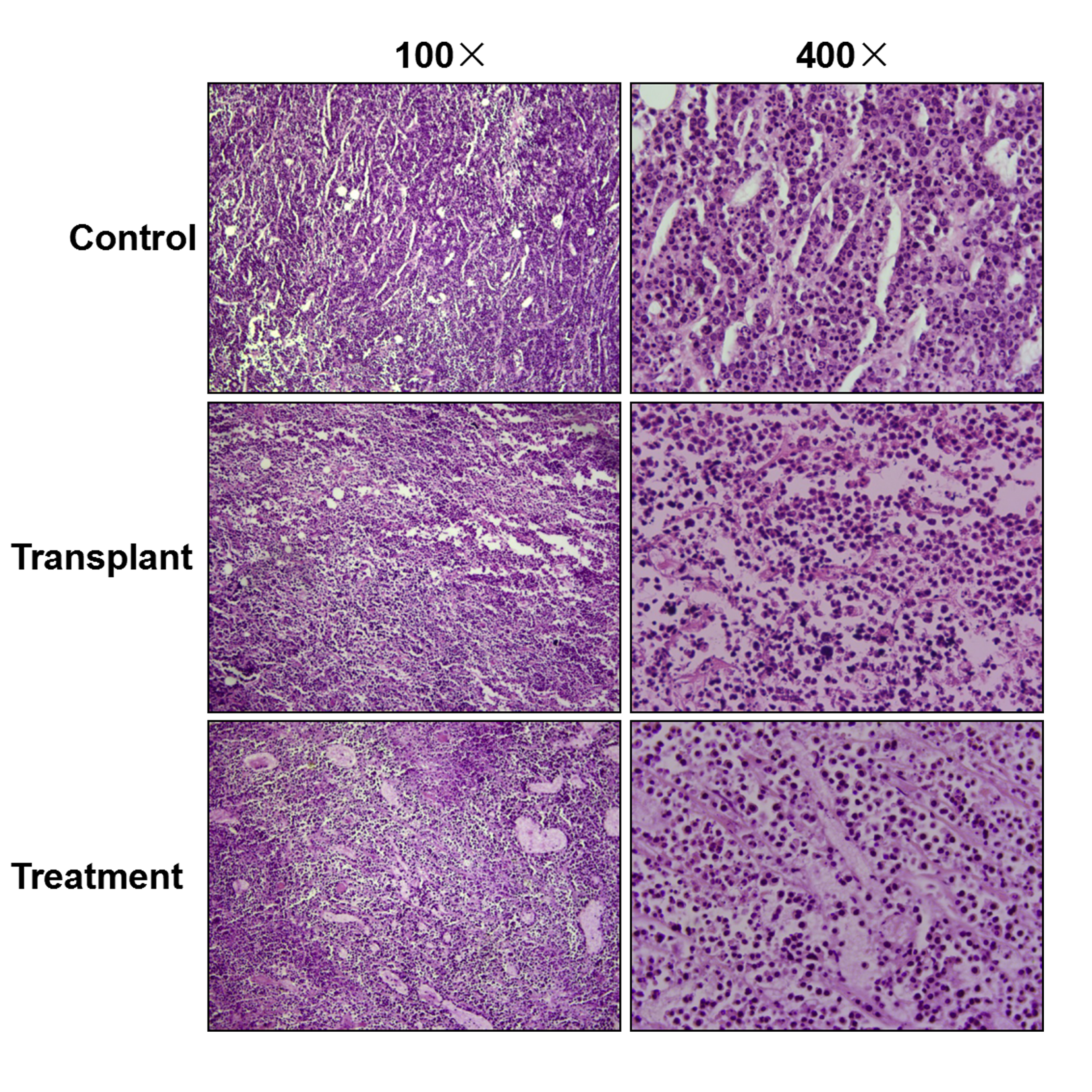


FIGURE S1: Comparison of changes in tumor histology. The tumor histology was determined by hematoxylin and eosin staining. Tumor tissues were harvested from control, transplant and treatment group. The control group is orally administrated with

0.2 ml 0.9% normal saline daily (n=10). The transplant group was challenged by 0.2 ml 0.9% normal saline daily after APBSCT (n=10). The treatment group was challenged with 0.2 ml Jiedu Xiaoluo decoction after APBSCT (n=10). Magnification: left (100x) and right (400x).


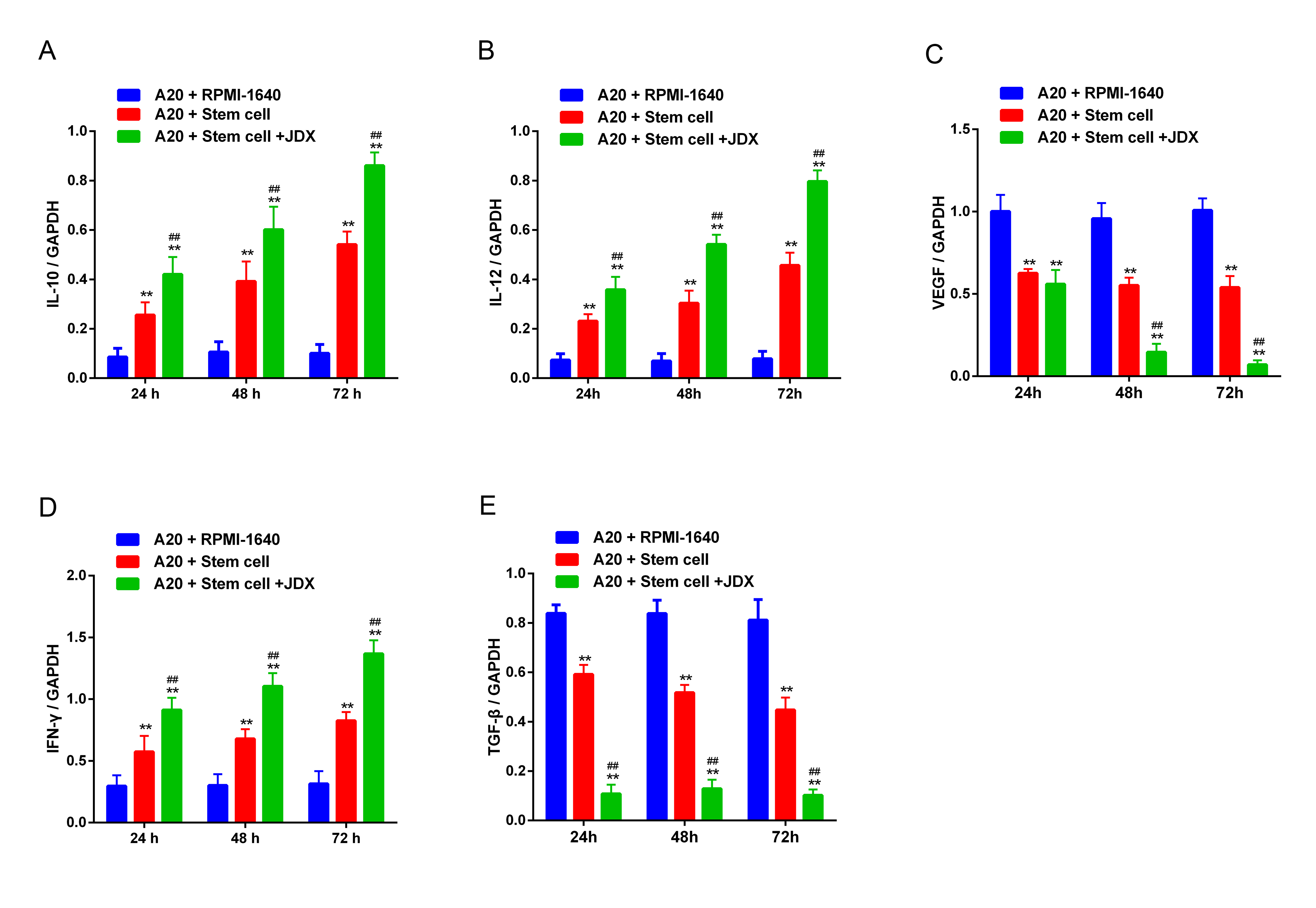


FIGURE S2: Semi-quantitative of inflammation-related protein in A20 cells. In the co-culture system, cells were treated with control serum and drug serum for 24 h, 48 h and 72 h. Semi-quantitative of IL-10 (A), IL-12 (B), VEGF (C), IFN- γ (D) and TGF-β (E) as analyzed by using ImageJ software.

Supplementary Figure S3:


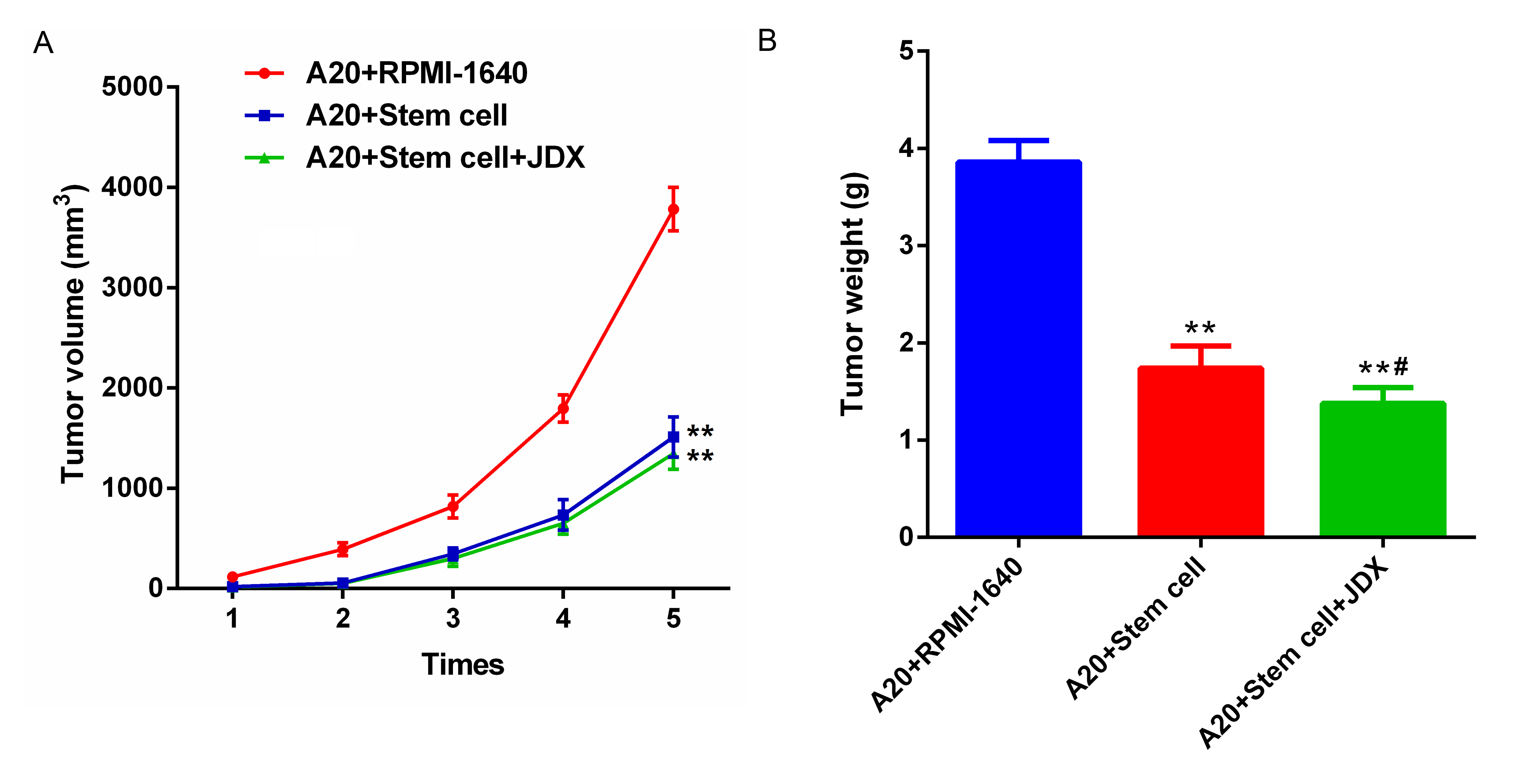


FIGURE S3: Establishment of xenograft model of A20 cells. (A) Tumor growth curve

data of tumor weight in the above three groups. * indicates control group VS co-culture with HSCs group and drug serum group. # indicates drug serum group VS co-culture with HSCs group. * or #, P < 0.05; ** or ##, *P* < 0.01.
